# Accurate assembly of multiple RNA-seq samples with Aletsch

**DOI:** 10.1093/bioinformatics/btae215

**Published:** 2024-06-28

**Authors:** Qian Shi, Qimin Zhang, Mingfu Shao

**Affiliations:** Department of Computer Science and Engineering, The Pennsylvania State University, University Park, PA 16802, United States; Department of Computer Science and Engineering, The Pennsylvania State University, University Park, PA 16802, United States; Department of Computer Science and Engineering, The Pennsylvania State University, University Park, PA 16802, United States; Huck Institutes of the Life Sciences, The Pennsylvania State University, University Park, PA 16802, United States

## Abstract

**Motivation:**

High-throughput RNA sequencing has become indispensable for decoding gene activities, yet the challenge of reconstructing full-length transcripts persists. Traditional single-sample assemblers frequently produce fragmented transcripts, especially in single-cell RNA-seq data. While algorithms designed for assembling multiple samples exist, they encounter various limitations.

**Results:**

We present Aletsch, a new assembler for multiple bulk or single-cell RNA-seq samples. Aletsch incorporates several algorithmic innovations, including a “bridging” system that can effectively integrate multiple samples to restore missed junctions in individual samples, and a new graph-decomposition algorithm that leverages “supporting” information across multiple samples to guide the decomposition of complex vertices. A standout feature of Aletsch is its application of a random forest model with 50 well-designed features for scoring transcripts. We demonstrate its robust adaptability across different chromosomes, datasets, and species. Our experiments, conducted on RNA-seq data from several protocols, firmly demonstrate Aletsch’s significant outperformance over existing meta-assemblers. As an example, when measured with the partial area under the precision-recall curve (pAUC, constrained by precision), Aletsch surpasses the leading assemblers TransMeta by 22.9%–62.1% and PsiCLASS by 23.0%–175.5% on human datasets.

**Availability and implementation:**

Aletsch is freely available at https://github.com/Shao-Group/aletsch. Scripts that reproduce the experimental results of this manuscript is available at https://github.com/Shao-Group/aletsch-test.

## 1 Introduction

The well-established high-throughput RNA sequencing (RNA-seq) technologies have been pivotal and instrumental in many biological and biomedical studies. The recent advancement in single-cell RNA-seq technologies further enables profiling gene activities at single-cell resolutions. One of the critical steps in RNA-seq analysis is the determination of full-length expressed transcripts from the RNA-seq reads, a computational problem commonly known as the *transcript assembly*. Over the decades, significant endeavors have been dedicated to this problem, including studying the mathematical formulations behind (Shao and Kingsford 2019; Dias *et al.* 2022; Khan *et al.* 2022) and developing practical tools, including Cufflinks (Trapnell *et al.* 2010), CLASS2 (Song *et al.* 2016), TransComb (Liu *et al.* 2016), StringTie series (Pertea *et al.* 2015; Kovaka *et al.* 2019), and Scallop series (Shao and Kingsford 2017; Zhang *et al.* 2022), to name a few.

Despite these efforts, transcripts assembled from a single sample or cell often remains incomplete or fragmented. One scenario is that if a junction were not sequenced, then there is little chance for a single-sample assembler to correctly recover it. This motivates *meta-assembly*—assembling full-length transcripts from multiple RNA-seq samples. Meta-assembly provides a promising opportunity to take advantage of shared information across various samples, to fill in the gaps, particularly the missing exons and junctions within individual samples, and to contribute to a more accurate and complete assembly. A trivial meta-assembly approach is a simple integration of RNA-seq reads from individual samples followed by calling a single-sample assembler; this method not only ignores the divergence of individual samples but also significantly increases the complexity of resolving the splicing variants, and hence performs poorly in practice. Several specific meta-assembly methods have been proposed, including MiTie (Behr *et al.* 2013), ISP (Tasnim *et al.* 2015), TACO (Niknafs *et al.* 2017), StringTie-merge, PsiCLASS (Song *et al.* 2019), and TransMeta (Yu *et al.* 2022). The two recent meta-assemblers, PsiCLASS and TransMeta, significantly pushed forward the state-of-the-art with their specific algorithmic advancements, but as far as we know, certain limitations remain. For example, PsiCLASS does not perform as well when sample experiences low coverage, which hinders its broad use for single-cell RNA-seq data; TransMeta requires an adequate number of individual graphs in one gene locus to confidently decompose the consensus graph, which may result in reduced accuracy on largely divergent samples. Meta-assembly is not as developed as single-sample assembly, creating a substantial gap that requires attention.

In addition to developing more sensitive algorithms, controlling falsely assembled transcripts is critical, as RNA-seq data are known to be notoriously noisy. Algorithms often produce incorrect transcripts on gene loci with complicated splicing variants, due to the lack of information to resolve ambiguity. While existing tools have developed heuristic criteria to eliminate spurious splice junctions (Yu *et al.* 2022), the reliance on fixed thresholds and rules often fails to accommodate the diverse scenarios with varying sequencing techniques and dataset distributions. Manually fine-tuning the large number of parameters provided by tools is too laborious and time-consuming to reach an optimum.

We introduce Aletsch, a new meta-assembler crafted for the precise assembly of multiple bulk or single-cell RNA-seq samples. Aletsch stands out by effectively harnessing information from multiple samples while retaining the distinct characteristics of each individual sample. It begins by constructing both individual and combined splice graphs. In these graphs, Aletsch “bridges” paired-end reads, leveraging the combined graph’s rich junction information to restore complete fragments. This approach not only refines each sample’s individual graph but also guides the graph decomposition with these full fragments. Aletsch extends the idea of “phase-preserving” (Shao and Kingsford 2017) in graph decomposition by factoring in edge supports from additional samples. This comprehensive approach allows Aletsch to generate a reliable set of candidate transcripts. Furthermore, we have integrated a random forest model, equipped with 50 well-designed features, to learn a scoring function that evaluates and ranks the assembled transcripts. With comprehensive experiments we demonstrate the model’s exceptional transferability across various chromosomes, datasets, and species.

## 2 Materials and methods

**Figure 1.**
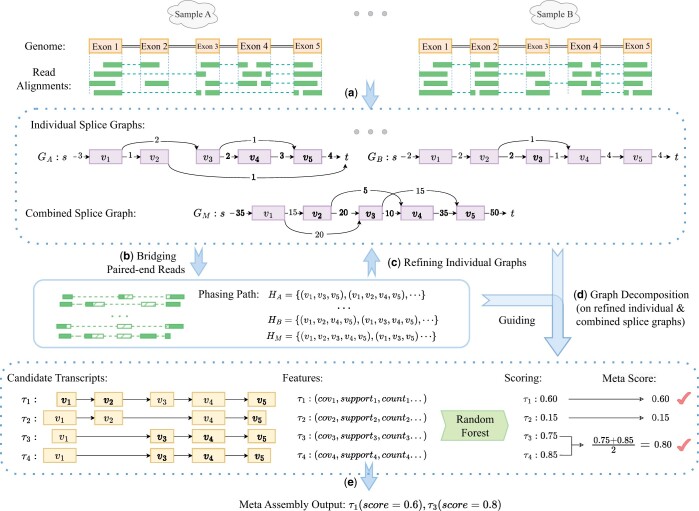
Workflow of Aletsch. (a) Constructing individual and combined splice graphs. Dashed green lines indicates junctions inferred from clipped reads. (b) Bridging paired-end reads to create enhanced phasing paths, shown as dashed green blocks. (c) Refining individual splice graphs. (d) Decomposing refined individual splice graphs and the combined graph, guided by enhanced phasing paths. (e) Grouping identical intron-chains (e.g. $\tau_{3}$ and $\tau_{4}$) and scoring candidate transcripts with a random forest model to generate the final meta-transcripts.

Aletsch takes alignments of a set of RNA-seq samples as input, and generates scored transcripts. It starts with constructing individual splice graphs for each sample, and a combined splice graph from all samples. Aletsch then uses an iterative bridging algorithm to “bridge” two mates of paired-end reads. The bridged reads are used to reconstruct more accurate individual and combined graphs, while also providing longer “phasing paths” for graph decomposition. Aletsch employs a new algorithm for decomposing both individual and combined graphs into candidate transcripts, guided by the enhanced phasing paths. Throughout this process, a comprehensive set of features are collected that characterize each candidate transcript. These features are then fed into in a random forest model to predict a score for each candidate transcript. A pipeline of Aletsch is illustrated in Fig. 1.

### 2.1 Construction and clustering of splice graphs

Splice graph is instrumental for studying alternative splicing and transcript assembly. A splice graph, formally written as G=(V,E,w), is a weighted acyclic graph, where *V* denotes exons or partial exons, *E* signifies splicing junctions, and w(·) represents coverage. Aletsch loads read alignments in each sample, and builds an *individual splice graph* for each gene locus. Specifically, splicing junctions are first extracted from the read alignments to form the edges *E*. These splicing positions also partition the reference genome, and consequently aid in identifying (partial) exons, which form the vertices *V*. The weight function w(·) assigns weights to both vertices and edges in *G*. The weight of a vertex v, w(v), is computed as the average coverage of the corresponding exon, and the weight of an edge e=(u,v), i.e. w(e) or w(u,v), is determined by the count of reads spanning the junction from exon *u* to exon *v*.

To make use of the shared signals across samples, Aletsch performs clustering over individual splice graphs, aiming to group splice graphs from different samples that correspond to the same gene locus. Aletsch computes the similarity of each pair of individual graphs $G_{i}$ and $G_{j}$, defined as $\left|S_{i}\cap S_{j}|/|S_{i}\cup S_{j}\right|$, where $S_{i}$ and $S_{j}$ are the set of splicing positions in $G_{i}$ and $G_{j}$, respectively. Aletsch then conducts single-linkage clustering starting from the highest similarity: a cluster stops growing when its size reaches a threshold (a parameter, which is often set as the number of samples).

Aletsch handles each cluster separately and independently. A combined splice graph, denoted as $G_{M}$, is constructed to represent the collective splicing and coverage information in a cluster. $G_{M}$ is constructed in the same way as individual graphs, but incorporates all reads used to build individual graphs in the cluster.

For either individual or combined graphs, vertices with in-degree of 0 are called *starting vertices*, represented as $V_{s}\subseteq V$, and those without-degree of 0 are called *ending vertices*, represented as $V_{t}\subseteq V$. We add a source vertex *s*, connecting to all starting vertices $u\in V_{s}$ with e=(s,u) of weight $w(s,u)=\sum_{(u,v)\in E}(w(u,v))$. Similarly, all ending vertices $v\in V_{t}$ are connected to a sink vertex *t*, with edge weight $w(v,t)=\sum_{(u,v)\in E}(w(u,v))$. Within these notations, transcripts are equivalent to *s*-*t* paths in the splice graphs.

### 2.2 Bridging

We focus on the assembly of paired-end bulk or single-cell RNA-seq data in this work. Bridging is the process of inferring full-length fragments, essentially reconstructing the unsequenced portion of paired-end reads. Successful bridging provides more accurate structures and weights of splice graphs, while also offering longer phasing paths for resolving complex splicing variants, both of which are crucial for improving transcript assembly. In previous work, we developed an algorithm for bridging paired-end RNA-seq reads in the context of assembling individual RNA-seq samples (Zhang *et al.* 2022). Specifically, we formulated bridging as seeking an optimal path in the underlying (individual) splice graph that connects the two ends of paired-end reads. An efficient dynamic programming algorithm was designed for this purpose. We demonstrated that this bridging technique significantly improves the assembly of both linear (Zhang *et al.* 2022) and circular (Zahin *et al.* 2024) transcripts.

**Figure 2.**
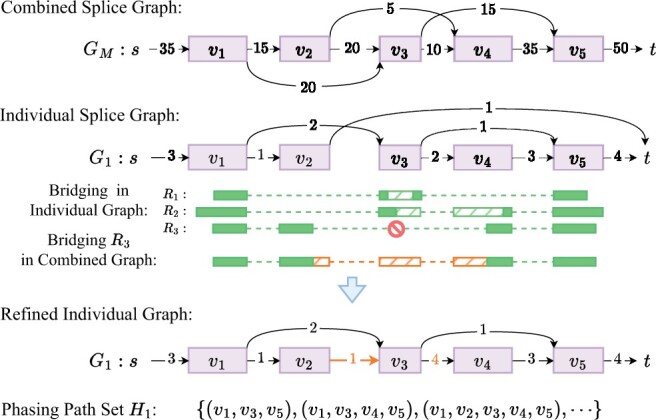
Bridging in Aletsch: Reads $R_{1}$ and $R_{2}$ are successfully bridged in graph $G_{1}$ (dashed green blocks). Read $R_{3}$ fails to bridge in $G_{1}$ due to the absence of a path from $v_{2}$ to $v_{4}$. But it can be bridged in combined graph $G_{M}$ (orange), restoring a missed junction, and updating edge weights in $G_{1}$. Bridged reads in $G_{1}$ form phasing path set $H_{1}$.

Using the above algorithm as a building block, here we develop a bridging framework for meta-assembly. First, paired-end reads are bridged independently for separate samples, using respective individual splice graphs. We then shift focus to reads that fail in this initial bridging. Such failures often indicate the incompleteness of individual splice graphs, typically due to missed junctions in corresponding samples (e.g. $R_{3}$ in Fig. 2). We therefore bridge these unbridged reads w.r.t. the combined splice graph, i.e. finding an optimal path in the combined splice graph that connects the two ends, using above algorithm. This stage of bridging will be successful if the missed junctions are sequenced in some other sample and appear in the combined graph. The bridged reads will then be used to refine the individual graphs, including restoring missed edges and correcting edge and vertex weights. See Fig. 2.

This new bridging framework represents one of the innovations of Aletsch that leverages information from multiple samples to boost meta-assembly. It is worth mentioning that Aletsch acknowledges that individual samples express differently: it prioritizes bridging within individual samples whenever feasible. Only when bridging fails, which strongly indicates incompleteness of individual splice graphs, does Aletsch “borrow” information from other samples. This cautious strategy keeps Aletsch from assembling transcript “artifacts” that mix junctions from different samples, striking a balance between maintaining sample-specific integrity and enhancing assembly accuracy.

We represent bridged fragments as paths in the underlying splice graphs, called *phasing paths*. These paths, along with the refined splice graph, are then piped into a subsequent graph-decomposition algorithm to obtain candidate transcripts. Phasing paths play a crucial role in guiding the graph-decomposition, particularly in complex branching scenarios.

### 2.3 Edge supports

We first construct a data structure that characterizes and stores the shared information across samples. This structure is integrated into the subsequent graph-decomposition algorithm. For each edge *e* in either an individual splice graph or the combined splice graph, we construct a size-*N* vector $M_{e}$, where $M_{e}[j]$ quantifies the “support” from $G_{j}$ to edge *e*, 1 ≤ j ≤ N, where *N* is the number of individual splice graphs in a cluster. Initially, $M_{e}[j]=0$ for all 1 ≤ j ≤ N. Below we show the calculation of $M_{e}[j]$ based on the type of edge *e*. Let *v* be a vertex. We use L(v) and R(v) to represent the left and right genomic coordinate of the (partial) exon corresponding to *v*. Similarly, for an edge e=(u,v), we use L(e) and R(e) to represent the left and right positions of *e*, i.e. L(e):=R(u) and R(e):=L(v). We categorize edges into three types: junction edge, adjacent edge, and starting edge, based on their structures.

#### 2.3.1 Junction edge 

**Figure 3.**
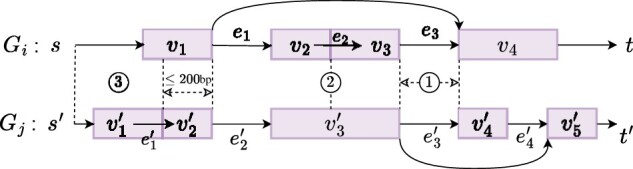
Types of edge support from splice graph $G_{j}$ to $G_{i}$: ① Junction edge. ② Adjacent edge. ③ Starting/ending edge.

We say edge e=(u,v) is a *junction edge* if *e* corresponds to a junction (i.e. L(e) + 1 < R(e) and u≠s and v≠t). We say junction edge e′ in $G_{j}$*supports e* if L(e′)=L(e) and R(e′)=R(e). If such e′ exists, we assign $M_{e}[j]=w(e\prime )$. In Fig. 3, ${e\prime }_{2}$ supports $e_{1}$, and ${e\prime }_{3}$ supports $e_{3}$.

#### 2.3.2 Adjacent edge

We say edge e=(u,v) is an *adjacent edge* if *u* is adjacent to *v* in its genomic positions, i.e. L(e) + 1=R(e). An adjacent edge e′ in $G_{j}$*supports e* if L(e′)=L(e) and R(e′)=R(e). If such e′ exists, we define $M_{e}[j]=w(e\prime )$. It is also possible that a vertex supports an adjacent edge. We define a vertex v′ in $G_{j}$*supports* an adjacent edge *e* if L(v′) ≤ L(e) + 1=R(e) < R(v′). In this case, we assign $M_{e}[j]=w(v\prime )$. As an example, ${v\prime }_{3}$ supports $e_{2}$ in Fig. 3.

#### 2.3.3 Starting/ending edge

We say edge e=(u,v) is a *starting edge* if u=s. The support mechanism for starting edges is more complex. To calculate $M_{e}[j]$, we locate position R(v) in $G_{j}$. If position R(v) locates within vertex v′ in $G_{j}$ and there exists a starting edge $e\prime =(s\prime ,v\prime )\in G_{j}$, where s′ is the source vertex of $G_{j}$, then, we say that starting edge e′ supports *e* and in this case we assign $M_{e}[j]=w(e\prime )$. In the absence of such a starting edge e′, we continue to examine if there exists an incoming adjacent edge (u′,v′) and a starting edge $e^{\prime \prime }=(s\prime ,u\prime )$, and if both exist, we also say $e^{\prime \prime }$ supports *e* and assign $M_{e}[j]=w(e^{\prime \prime })$. We impose a limitation on such left-extension that R(v)−R(u′) should not exceed a threshold (200 base pairs by default). In Fig. 3, starting edge $\left(s\prime ,v_{1}^{\prime }\right)$ supports edge $\left(s,v_{1}\right)$. The calculation of edge support for any ending edge *e* mirrors that of starting edges but extending to the right. We say edge e=(u,v) is an *ending edge* if v=t. In Fig. 3, no ending edges in $G_{j}$ supports edge $\left(v_{4},t\right)$.

### 2.4 Graph decomposition

Aletsch implements a novel graph-decomposition algorithm that inputs a weighted splice graph G=(V,E,w), edge supports $M_{e}$ for each e∈E, and a set of phasing paths *H*. It produces a set of *s*-*t* paths *P* termed as *candidate transcripts*. This algorithm is consistently applied to each individual splice graph and the combined splice graph. The framework of this algorithm is to iteratively decompose vertices of *G* until it is reduced to a collection of parallel *s*-*t* paths. We follow two principles in decomposing a single vertex. The first one is *phase-preserving*: any phasing path h∈H must appear entirely in at least one *s*-*t* path p∈P. This principle is based on the understanding that phasing paths, derived from RNA fragments, should be segments of some expressed transcripts. We achieve phase-preserving by not breaking any phasing paths during decomposition. In the absence of phasing paths, we use the supporting information (i.e. $M_{e}$) to guide the decomposition—this is the second principle.

**Figure 4.**
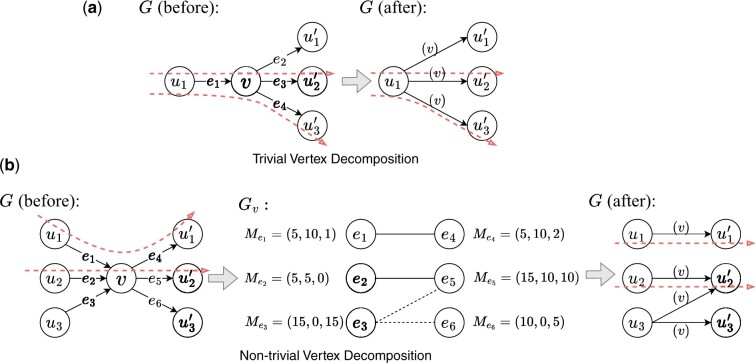
Examples of vertex decomposition. Red dashed arrow lines represent the phasing paths. In (b), each $M_{e}$ has three entries, representing edge support from three samples in the gene locus. Isolated vertices in the bipartite graph $G_{v}$ are resolved from left to right. For the isolated vertex $e_{3}$ on the left side, $e_{5}=\arg\max_{e_{o}\in \{e_{4},e_{5},e_{6}\}}(\sum_{1\leq j\leq 3}M_{e_{3},e_{o}}[j])$. Accordingly, we add an edge between $e_{3}$ and $e_{5}$ in $G_{v}$, shown as a black dashed line.

We design two subroutines for decomposing a single vertex, depending on its type. A *trivial* vertex in V∖{s,t} is defined as having either in-degree of 1 or out-degree of 1. The procedure for decomposing a trivial vertex *v* is straightforward—see Fig. 4a. For trivial vertex *v*, given an in-edge $e_{i}$ and an out-edge $e_{o}$, we create a new edge $\left(e_{i},e_{o}\right)$ after decomposing *v*. The edge support for this new edge is calculated as: $M_{e_{i},e_{o}}[j]=\min\{M_{e_{i}}[j],M_{e_{o}}[j]\}$, for every 1 ≤ j ≤ N.

To decompose a non-trivial vertex *v*, we construct a bipartite graph $G_{v}=(V_{i}\cup V_{o},E_{v})$, where $V_{i}$ and $V_{o}$ correspond to the in-edges and out-edges of *v* in *G*, respectively. If there exists a phasing path *h* that threads through $e_{i}$, *v*, and $e_{o}$ in *G*, we add an undirected edge $\left(e_{i},e_{o}\right)$ into the bipartite graph $G_{v}$. The edge supports for $\left(e_{i},e_{o}\right)$ is calculated as $M_{e_{i},e_{o}}[j]:=\min\{M_{e_{i}}[j],M_{e_{o}}[j]\}$ for each 1 ≤ j ≤ N. There might be “isolated” vertices (degree of 0) in $G_{v}$, i.e. they are not threaded by any phasing paths, as shown in Fig. 4b. We employ edge supports to add edges for them in $G_{v}$. Specifically, for an isolated vertex *e* (assuming $e\in V_{i}$ without loss of generality), we calculate $e_{o}^{*}:=\arg\max_{e_{o}\in V_{o}}(\sum_{1\;\leq \;j\;\leq \;N}M_{e,e_{o}}[j])$, and then, add an edge $\left(e,e_{o}^{*}\right)$ to $G_{v}$. This procedure is applied to all isolated vertices in $G_{v}$.

The decomposition of *G* follows the structure of $G_{v}$: every edge $\left(e_{i},e_{o}\right)$ in $G_{v}$ becomes a new edge $\left(e_{i},e_{o}\right)$ in *G* after decomposing *v*. Additionally, the decomposed vertex *v* is recorded on these new edges with an auxiliary data structure, allowing for the reconstruction of the vertices list for the resulting *s*-*t* paths.

The above algorithm extends the graph-decomposition algorithm developed in Shao and Kingsford (2017), from which Aletsch adapts its iterative framework and the “phase-preserving” principle. We also reuse the weight-updating procedures in Scallop to calculate the weight w(e) for each newly created edge *e*; at the end of the decomposition, when *G* becomes a set of parallel *s*-*t* edges, this weight represents the inferred abundance of the corresponding candidate transcript. The innovation of this algorithm lies in its utilization of signals of multiple samples (i.e. edge supports) to guide the decomposition. Aletsch gives higher priority to phasing information associated with each individual graph, and only when such information is not available (i.e. isolated vertices), the information from other samples are employed. This strategy again reduces the chance for Aletsch to assemble transcript artifacts.

It is worth noting that, for each *s*-*t* edge *e* at the end, if $M_{e}[j]\;>\;0$, it suggests that the *s*-*t* path corresponding to *e* also exists as a path in the splice graph of the *j*-th sample (this *s*-*t* path may or may not be assembled in the *j*-th sample, though). Hence, both $\left|\{j|M_{e}[j]\;>\;0,1\;\leq \;j\;\leq \;N\}\right|$ and $\sum_{1\;\leq \;j\;\leq \;N}M_{e}[j]$ are informative features that quantify how much other samples support this candidate transcript *e*. These features, together with others such as w(e), will be used in the subsequent machine-learning model for scoring.

### 2.5 Features

It is of great interest to assign a confidence score to assembled transcript, indicating how likely it is to be correct. Traditionally, the inferred abundance serves this purpose, given its observed positive correlation with correctness. We explore a more precise scoring function through machine learning. Rooted in our deep understanding and extensive experience on transcript assembly, we have engineered 50 features to comprehensively characterize an assembled candidate transcript, ranging from its decomposition to its interaction with other transcripts and graphs. We categorize them below; a detailed description with intuitions is in Supplementary Note S1.

1. *Abundance Features*. This category includes the inferred abundance, number of supporting samples and their total weights, “bottleneck” weights and sample supports, and so forth.
2. *Graph Structural Features*. This includes the number of vertices and junctions in the related individual splice graph and the combined splice graph, and so forth.
3. *Boundary Features.* One major challenge in transcript assembly is the accurate prediction of starting and ending sites. We use features such as the supports of a boundary from other samples. They are particularly useful for single-cell RNA-seq data, where transcripts are more likely to be partially sequenced, which leads to false boundaries.
4. *Intron Features.* The differentiation of transcripts with intron retentions from contaminated reads in intron regions presents another challenge. Features including the “competitiveness” of intron coverage and surrounding junctions are informative to distinguish them.

### 2.6 Scoring and meta-assembly

We trained a random forest to score candidate transcripts, each represented by a length-50 feature vector. The model is configured with 100 estimators and a maximum depth of 20, performing binary classification to yield a probability score between 0 and 1 for each candidate transcript.

It is common for “identical candidate transcripts” to be assembled from different samples, or from an individual graph and the combined graph. Here, we define two candidate transcripts as identical if they share the same intron-chain, a definition widely accepted. We aggregate identical candidate transcripts into a single “meta-transcript.” The score of a meta-transcript is calculated as the average score of its consisting candidate transcripts (Fig. 1). We recommend discarding meta-transcripts with a score below 0.2. Aletsch sets a default threshold of 0.5, which users can adjust to balance recall and precision. Aletsch outputs the set of meta-transcripts with their scores, consistent with other meta-assemblers.

## 3 Results

### 3.1 Experimental setup

We compare Aletsch with two existing meta-assemblers, TransMeta (v.1.0) and PsiCLASS (v1.0.3), and two additional meta-assembly pipelines based on single-sample assemblers, StringTie2-merge (v2.2.1) which we refer to as ST2Merge for short, and Scallop2 (v1.1.2) combined with Taco (v0.7.3), referred to as SC2TACO. All methods take a set of aligned RNA-seq samples or cells as input and produce a set of (scored) meta-transcripts. When the RNA-seq data is non-synthetic which we often do not know the true expressed transcripts, the commonly used ground-truth for evaluation is reference annotation. A multi-exon meta-transcript is considered as a “match” if its intron-chain exactly matches with a transcript in the reference annotation. Two metrics can then be calculated with tool GffCompare (Pertea and Pertea 2020): *precision*, defined as the ratio between the number of matched and the total number of predicted meta-transcripts, and *recall*, defined as the ratio of the number of matched meta-transcripts to the total number of transcripts in the annotation. To conclude when one method has a higher recall but lower precision, or vice versa, it is common to draw the precision-recall curves (PRC) with a varying parameter. The parameter used for this purpose is the inferred confidence score for Aletsch, and the predicted abundance for other methods (Supplementary Note S2). In this way, the performance of different methods over a broader range of parameters can also be compared. Experimental results shown in the main text are all conducted using the Ensembl annotation. Results using the RefSeq annotation are available in Supplementary Fig. S7 and Supplementary Tables S1 and S2.

Our experiments were conducted on eight real datasets, including both bulk and single-cell RNA-seq data, detailed in Table 1 and Supplementary Tables S6 and S7. The comparison of runtime and memory is given in Supplementary Note S3 and Supplementary Tables S4 and S5. Since Aletsch has a machine-learning module for scoring, we pay close attention to the model’s transferability to different datasets or even to different species. Therefore, we partition the eight datasets into three groups: the models are trained using G1, including four human datasets; G2 serves to show the models’ performance on different human datasets, and G3 is used to show its performance on a new species. Note that G1 includes both bulk and single-cell datasets. Integrating both is challenging as they exhibit different properties such as sample size and read coverage. Aletsch addressed this challenge, thanks to its comprehensive feature-engineering and normalizations on particular features.

**Table 1.** Summary of real datasets.

Group	Name	Protocol	Species (tissue)	Size	Accession ID	References
G1	BK-H1	Illumina paired-end	Human (skin, etc.)	10 samples	SRR307903, etc.	Shao and Kingsford (2017)
	BK-H2	Illumina paired-end	Human (liver)	73 samples	PRJNA57230	Song *et al*. (2019)
	SC-H1	Smartseq3	Human (kidney)	100 cells	E-MTAB-8735	Hagemann-Jensen *et al*. (2020)
	SC-H2	Smartseq3-Xpress	Human (blood)	1066 cells	E-MTAB-11452	Hagemann-Jensen *et al*. (2022)
G2	BK-H3	Illumina paired-end	Human (kidney)	12 samples	PRJNA489891	Yu *et al*. (2022)
	SC-H3	Smartseq3	Human (kidney)	92 cells	E-MTAB-8735	Hagemann-Jensen *et al*. (2020)
G3	BK-M1	Illumina paired-end	Mouse (hippocampi)	44 samples	PRJEB18790	Song *et al*. (2019)
	SC-M1	Smartseq3	Mouse (tail)	369 cells	E-MTAB-8735	Hagemann-Jensen *et al*. (2020)

We give a name for each dataset in the second column, where “BK” and “SC” stand for bulk and single-cell.

We train two models Aletsch-Chr1 and Aletsch-ChrAll. To train Aletsch-Chr1, we extract the RNA-seq reads from G1 datasets aligned to chromosome 1. Aletsch is run on these reads to produce candidate transcripts, each equipped with a feature vector of size 50. These candidate transcripts are then labeled, using a given annotation, where a candidate transcript gets labeled as “1” if it matches the intron chain of an existing transcript in the annotation, and “0” otherwise. These training samples, each consisting of the feature vector plus the label, are then used to train a random forest model. Aletsch-ChrAll is trained similarly to Aletsch-Chr1, except that all reads in the G1 datasets are used.

We note that Aletsch-Chr1 “sees” the transcripts of chromosome 1 in the annotation, while Aletsch-ChrAll sees all transcripts in the annotation. Since the same annotation also serves as the ground-truth to evaluate the accuracy of the trained model, there is potential risk of “data-leaking.” We emphasize that the features Aletsch uses are the quantification of either transcripts’ abundances or structures, without revealing any genomic coordinates; hence, data-leaking is impossible. But still, we carefully designed the experiments to prove that Aletsch learns the intrinsic characterizations of true transcripts. To do so, we test the accuracy of Aletsch-Chr1 on other chromosomes (excluding chromosome 1), as annotated transcripts for these chromosomes are never seen by Aletsch-Chr1. Below, we will show that Aletsch-Chr1 substantially outperforms existing meta-assemblers on other chromosomes and that the accuracy of Aletsch-Chr1 matches the accuracy of Aletsch-ChrAll. These jointly testify that no data leaks occur in the training process and both models can be safely used.

### 3.2 Results on other chromosomes of G1

**Figure 5.**
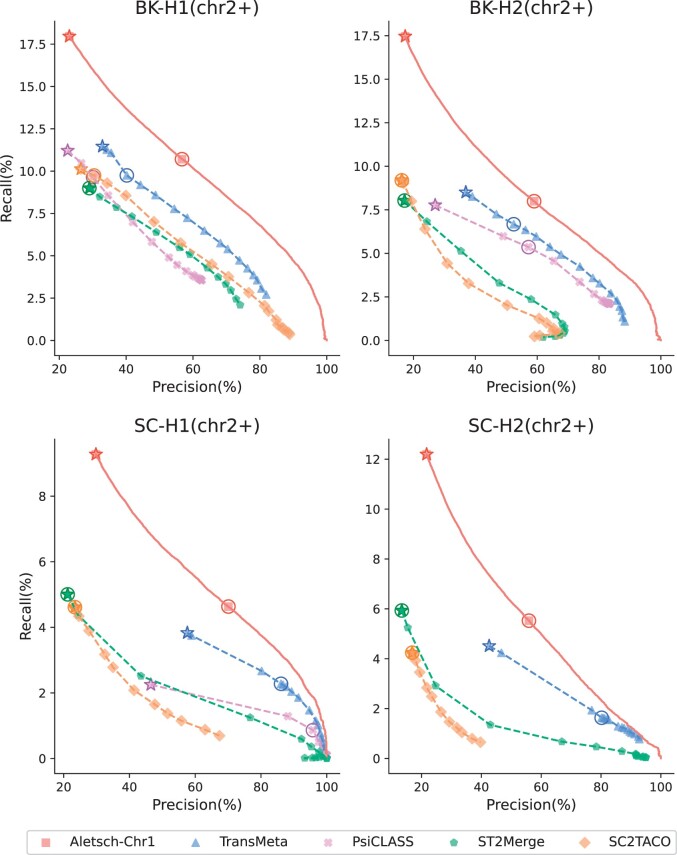
Comparison of PRCs of different assemblers on G1. Circled points indicate each tool’s default settings, while starred points denote the maximum recall achieved with the coverage filter disabled.

We first compare Aletsch-Chr1 with other meta-assembly methods on G1, but for chromosomes other than the first (termed chr2+). Their PRCs are given in Fig. 5. Note that Aletsch-Chr1’s curve is notably higher on all four datasets, showing that Aletsch outperforms other meta-assemblers.

To quantify the improvement, we measure the area under the PRC. Since the ranges of precision or recall of different methods vary, we compare Aletsch with each of the other methods by locating the shared range, either by recall or by precision, and calculate the partial area under the PRC (pAUC). The detailed results are given in Tables 2 and 3. We observe that the improvement of Aletsch over other methods is substantial. On G1 datasets, the improvement over the second-best method TransMeta, ranges from 9.2% to 31.6% when the pAUC is constrained by recall. The improvement is even more pronounced when the pAUC is constrained by precision (39.6%–50.6%); this is because Aletsch is able to assemble more matched transcript at the cost of the same amount of precision.

**Table 2.** Comparison of pAUC (%, constrained by *recall*).

Dataset	Aletsch versus TransMeta			Aletsch versus PsiCLASS			Aletsch versus ST2Merge			Aletsch versus SC2TACO		
	Alet.	Tran.	Δ %	Alet.	PsiC.	Δ %	Alet.	ST2M.	Δ %	Alet.	SC2T.	Δ %
BK-H1(chr2+)	6.74	5.12	31.6	5.81	3.13	85.5	5.86	3.74	56.9	8.30	5.87	41.4
BK-H2(chr2+)	5.95	4.97	19.8	4.53	3.31	36.9	6.57	3.43	91.5	7.17	3.13	129.4
SC-H1(chr2+)	3.33	3.05	9.2	2.09	1.78	17.7	4.42	2.65	66.7	3.47	1.53	126.6
SC-H2(chr2+)	2.91	2.53	14.9	N/A	N/A	N/A	4.47	1.99	123.9	2.86	0.89	220.5
BK-H3(chr1)	7.84	6.87	14.1	3.68	2.94	25.0	9.65	7.12	35.5	6.68	5.10	30.9
BK-H3(chr2+)	7.00	6.14	13.9	3.58	2.82	27.1	7.23	5.47	32.2	8.34	6.55	27.3
SC-H3(chr1)	3.97	3.57	11.1	3.01	2.43	23.9	5.16	3.01	71.5	0.84	0.27	207.5
SC-H3(chr2+)	3.32	3.05	8.9	2.17	1.83	18.3	4.38	2.59	69.0	3.45	1.54	123.3
BK-M1	7.12	6.34	12.3	5.10	4.26	19.7	10.64	8.18	30.0	12.85	7.28	76.5
SC-M1	6.16	5.46	12.8	5.07	3.55	42.9	8.65	5.16	67.5	6.60	3.36	96.6

Aletsch-Chr1 was used for G1 and G2 datasets; and Aletsch-ChrAll for G3. PsiCLASS experienced segment-fault on SC-H2.

**Table 3.** Comparison of pAUC (%, constrained by *precision*).

Dataset	Aletsch versus TransMeta			Aletsch versus PsiCLASS			Aletsch versus ST2Merge			Aletsch versus SC2TACO		
	Alet.	Tran.	Δ %	Alet.	PsiC.	Δ %	Alet.	ST2M.	Δ %	Alet.	SC2T.	Δ %
BK-H1(chr2+)	5.23	3.57	46.6	5.38	2.83	90.3	5.29	2.68	97.4	6.66	3.51	90.1
BK-H2(chr2+)	3.89	2.79	39.6	4.97	2.97	67.2	5.71	2.09	173.2	5.76	1.72	234.1
SC-H1(chr2+)	1.57	1.09	44.5	2.28	0.84	171.5	4.48	1.59	182.4	3.18	0.92	246.0
SC-H2(chr2+)	2.00	1.33	50.6	N/A	N/A	N/A	5.15	1.23	317.2	2.39	0.43	450.2
BK-H3(chr1)	5.45	4.39	24.0	4.42	3.59	23.0	8.86	5.29	67.5	6.06	3.92	54.6
BK-H3(chr2+)	4.79	3.90	22.9	4.03	3.18	26.5	4.35	2.52	72.3	5.70	3.51	62.5
SC-H3(chr1)	2.09	1.29	62.1	3.90	1.42	175.5	5.54	1.86	197.5	0.61	0.26	130.4
SC-H3(chr2+)	1.53	1.06	43.8	2.35	0.89	165.0	4.42	1.54	187.0	2.98	0.91	227.9
BK-M1	6.71	5.93	13.1	6.29	5.35	17.5	7.39	4.36	69.7	6.60	3.20	106.0
SC-M1	3.94	3.12	26.2	6.25	2.18	186.0	7.75	3.24	139.3	7.15	2.57	178.2

Aletsch-Chr1 was used for G1 and G2 datasets; and Aletsch-ChrAll for G3. PsiCLASS experienced segment-fault on SC-H2.

The success of Aletsch-Chr1 suggests that the model learned from one chromosome can be transferred to other chromosomes, proving that the model effectively captures the intrinsic features of true transcripts. Its high accuracy on both bulk and single-cell datasets also illustrates the feasibility of developing a versatile model for multiple types of datasets.

Many cells in the SC-H2 dataset have low read coverage, and it is expected that some junctions in these cells are not sequenced. This makes methods based on single-sample assemblers almost impossible to assemble correctly. Aletsch is able to recover the missed junctions from other cells/samples. Aletsch also decomposes the combined graph, another mechanism to rescue such transcripts. Note that whether a transcript gets assembled in both the individual and combined graphs will be recorded as features which will eventually be reflected in its confidence score. Aletsch is therefore still accurate in assembling single-cell datasets with low read coverage. For example, the improvement of Aletsch over ST2Merge and SC2TACO on this dataset is 123.9% and 220.5% when measured with pAUC constrained by recall.

### 3.3 Results on G2

**Figure 6.**
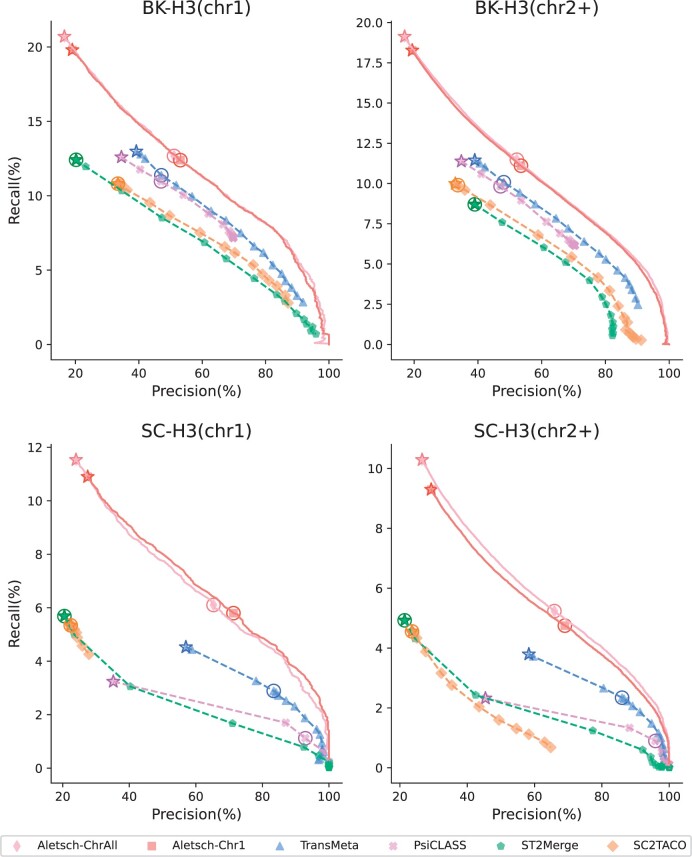
Comparison of PRCs of different assemblers on G2. Circled points show default settings; starred points indicate filtering disabled.

We investigate the performance of Aletsch on G2 that are not touched in the training. We separate chromosome 1 and other chromosomes. Figure 6 gives the PRCs of Aletsch-Chr1, Aletsch-ChrAll, and other methods. Aletsch substantially outperforms other methods on all datasets, demonstrating its transferability to different datasets. Another two key observations are that Aletsch-Chr1 shows no obvious difference between chr1 and the other chromosomes, and that Aletsch-Chr1 and Aletsch-ChrAll exhibit nearly identical performance. This consistency firmly proves that Aletsch has learned the intrinsic characteristics to select true transcripts. Otherwise, one would observe a significant performance drop on other chromosomes and a significant performance boost when using Aletsch-ChrAll.

### 3.4 Results on G3

**Figure 7.**
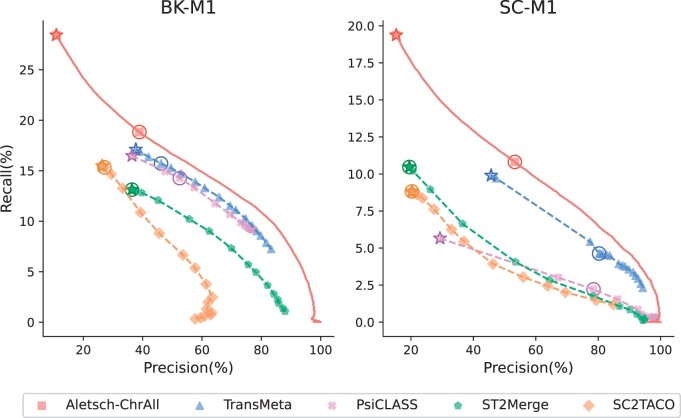
Comparison of PRCs of different assemblers on G3. Circled points show default settings; starred points indicate filtering disabled.

We compare the performance of different meta-assemblers on G3, consisting of two mouse datasets. We are particularly interested in if a model learned from human can achieve similar improvement on a different species. The PRCs are given in Fig. 7, in which Aletsch-ChrAll is used. Again Aletsch achieves superior accuracy consistently, indicating an remarkable transferability across different species.

### 3.5 Results on simulations

One of the major challenges for meta-assembly is that the input samples/cells may or may not express similar sets of transcripts. We investigate the performance of different methods at different levels of “similarity” using simulations. We simulated three datasets using Polyester (Frazee *et al.* 2015), namely Polyester-Sim90, Polyester-Sim50, and Polyester-Sim10, each consisting of 30 samples. In simulation, we first randomly picked 101 453 multi-exon transcripts across 9985 gene loci from the Ensembl annotation. The ground-truth of each sample is created by randomly picking *p*% transcripts from this set, where p=10,50,90, corresponding to the three datasets. Therefore, the ground-truth transcripts in the Polyester-Sim90 samples will be more likely to be shared. Aletsch was trained on a separate simulated dataset with p=50 from a ground-truth that does not share any gene used in simulating the 3 testing datasets.

**Figure 8.**
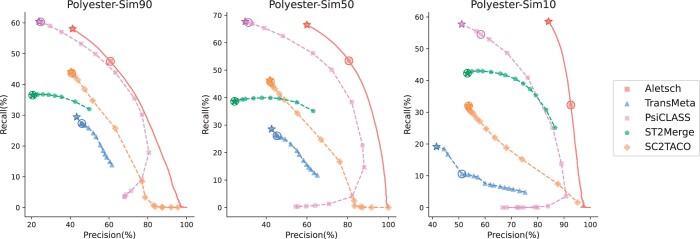
Comparison of PRCs of different assemblers on simulated datasets. Circled points show default settings; starred points indicate filtering disabled.

The PRCs are given in Fig. 8. TransMeta and PsiCLASS performed quite differently on these simulated datasets compared to real datasets. Aletsch, however, adapted to new distributions and outperformed the others. Specifically, Aletsch achieved pAUC, constrained by recall, 57.7%–78.3% higher than TransMeta, 10.2%–17.4% higher than PsiCLASS, 17.5%–84.6% higher than ST2Merge, 29.9%–42.8% higher than SC2TACO.

As sample similarity decreased, ST2Merge’s performance became closer to that of PsiCLASS, while TransMeta’s results deteriorated. This is likely attributed to the difficulty in achieving “consensus” in TransMeta or making confident “voting” decisions in PsiCLASS under low similarity. Aletsch, in contrast, recognizes the diversity in the expressed transcript across samples and avoids enforcing consensus. This strategy allows Aletsch to achieve much improved accuracy in cases like Polyester-Sim10, where samples show high variability.

## 4 Discussion

We introduced Aletsch, a new meta-assembler for bulk and single-cell RNA-seq data. Aletsch achieved superior accuracy by fully taking advantage of shared information across multiple samples. It’s flexible, allowing easy retraining for new sequencing protocols or tissue-specific datasets. For under-explored species lacking annotations, we recommend training on phylogenetically similar species, thanks to Aletsch’s proven transferability across datasets and species.

Aletsch is currently tailored for short-read bulk and single-cell RNA-seq data. Recent advancements in long-read RNA-seq technologies offer improved capabilities for detecting full-length isoforms. Extending Aletsch to concurrently assemble both short-reads and long-reads represents a formidable task that we intend to explore. Despite the emergence of long-read technologies, Aletsch remains highly impactful, as short-read RNA-seq data continue to serve as the *de facto* standard and are widely utilized. Moreover, a large volume of short-read RNA-seq data has been deposited, making Aletsch applicable for enhanced analysis and the potential discovery of novel biological insights. On the other hand, identifying full-length isoforms from long-read RNA-seq data remains a challenge, particularly in cases where annotation is unavailable, primarily due to difficulties in accurately determining splicing junctions (Pardo-Palacios *et al.* 2021). This limitation is one reason why we did not opt for long-read datasets as the ground truth for evaluation. Meta-assembly that harnesses the strengths of both short-read and long-read RNA-seq data, which we plan to extend Aletsch for, holds promise for significantly advancing isoform reconstruction.

Currently, the score for a meta-transcript is calculated by averaging the scores of its corresponding identical transcripts. This approach proves effective for our scenario, as we observe that the scores for the candidate identical transcripts tend to cluster within a narrow range. This phenomenon is mainly because of the shared “meta-coverage” across all identical transcripts. We recognize that directly learning a score for each meta-transcript is an intriguing machine-learning task and worth independent exploration.

Besides a random forest, we also experimented with training a sequence model, specifically an LSTM, for path embedding. While LSTMs excel in processing time series data, our efforts to use them for binary classification of transcripts (determining the correctness of a transcript) were not entirely successful: the model struggled to capture the relationship between adjacent edges in an *s*-*t* path. Although our current random forest model performed well on existing features, we plan to investigate advanced embedding techniques to hopefully replace hand-crafted features.

## Supplementary Material

btae215_Supplementary_Data

## Data Availability

All real RNA-seq datasets used in this study are publicly available; accession IDs are provided in Table 1 and Supplementary Tables S6 and S7. For the Smartseq3 protocol, data can be accessed at E-MTAB-8735. We used source HEK293T with 192 human cells, divided into the SC-H1 dataset (100 cells) and the SC-H3 dataset (92 cells), and source Mouse-Fibroblast with 369 mouse cells for the SC-M1 dataset. For the Smartseq3-Xpress protocol, data are available at E-MTAB-11452, with source PBMCs_run2 used to create the SC-H2 dataset.
