# Supplementary material for: Accurate assembly of multiple RNA-seq samples with Aletsch
Source: Bioinformatics. 2024 Jun 28;40(Suppl 1):i307–17. doi: 10.1093/bioinformatics/btae215 (PMC11211816; doi:10.1093/bioinformatics/btae215)
Supplement: btae215_Supplementary_Data [file btae215_supplementary_data.zip › btae215_Supplementary_Data/Shao.262.sup.1.pdf]

Supplementary Materials for
"Accurate Assembly of Multiple RNA-seq Samples with Aletsch"

Qian Shi[1], Qimin Zhang[1], and Mingfu Shao[1,2,*]

[1]Department of Computer Science and Engineering, School of Electronic Engineering and Computer Science, The Pennsylvania State University
[2]Huck Institutes of the Life Sciences, The Pennsylvania State University

January 25, 2024

List of Supplementary Notes

1 Features Description 2
2 Threshold Setting for Precision-Recall Curves 4
3 Comparison of Runtime and Memory 5

List of Supplementary Figures

1 Illustrative Examples for Two Features. 3
2 Coverage Curves of Meta-assemblers 6
3 How Number of Samples Impact on Meta-Assembler Performance 7
4 Performance of Merged Input Alignments on Single-Sample Assemblers 8
5 Case Study 1: Performance Comparison at Gene RPL31 9
6 Case Study 2: High-Scored Mismatch of Aletsch at Gene POLR2H 10
7 Precision-Recall Curves Evaluated on RefSeq Annotation 11

List of Supplementary Tables

1 pAUC Constrained by Recall Evaluated on Refseq Annotation 12
2 pAUC Constrained by Precision Evaluated on Refseq Annotation 13
3 Isoform-level and Gene-level Analysis of Assemblers 14
4 Running Time Comparison 15
5 Memory Usage Comparison 16
6 Accession IDs of BK-H1 dataset. 17
7 Accession IDs of BK-M1 dataset. 18

*Correspondence should be addressed to mxs2589@psu.edu.

Supplementary Note 1: Features Description

Features are generated for every occurrence of a transcript τ, across different samples. Consider τ derived from an individual graph $G = (V, E, w)$ with source s and sink t. The features include:

(Shown in brackets are the headers of the Aletsch's feature table.)

Abundance Metrics

- Coverage(cov, cov2):
  - cov: Meta expression level of τ in the meta-assembly.
  - cov2: Expression level of τ in an individual sample. Summation of "cov2" across all instances equals "cov".
- Count (count1, count2):
  - count1: Estimated number of samples potentially outputting τ, calculated from the number of positive entries in the final edge support vector of τ.
  - count2: Actual number of samples outputting the same intron chains as τ.
- Support: The sum of all weights in the final edge support vector of τ.
- Bottleneck Abundance (seq_min_wt, etc.):
  - seq_min_wt, seq_min_cnt, seq_min_support: Measures the weakest junctions in τ in terms of junction weight, count, and support.
  - seq_min_ratio: Specifically measures the branching ratios at each vertex in the splice graph of τ. For an edge $e = (u, v) \in E_\tau$, where E_τ is the set of edges consisting τ, the incoming weight ratio of v in τ is defined as $w(e)$ divided by the total weight of incoming edges to v. Similarly, the outgoing weight ratio of u in τ is $w(e)$ divided by the total weight of outgoing edges from u. "seq_min_ratio" is the minimum of these ratios across all vertices in τ.
- Max Abundances (seq_max_wt, seq_max_ratio, etc.): Similar to bottleneck abundance, but focuses on maximum for each metric.
- (gr_reads): Number of aligned reads in G.
- (confidence): Ratio of decomposition confidence, particularly in scenarios where an edge cannot be extended by phasing paths. See section "Methods - Graph Decomposition".

Graph Structural Metrics

- V, E : Number of vertices and edges in splice graph G.
- V_t: Total count of all partial exons in transcript τ.
- E_t: Total count of both splicing and adjacent edges constituting τ. Adjacent edges connect closely neighboring partial exons. $E_t = V_t - 1$.
- (num_exons): Total number of exons in τ.
- (gr_subgraphs): Number of individual graphs in the gene locus.
- (junc_ratio): Ratio of splicing edges to total edges($|E_t|$) in $t =$.
- (uni_junc): Number of unique junctions in τ not present in any other transcripts from the same graph G.

Boundary Features

- Start/End Loss (start_loss1, end_loss1, etc.): Aggregated weights of junctions from other individual graphs that can extend τ further left or right. Refer to “Examples” for details.
- Start/End Abundance (start/end_weight, etc.): Measures the abundance of starting and ending edges, providing insights into the transcript's boundaries.
- Unbridge Reads (unbridge_start_coming_count, etc.): Number and scores of unbridged reads at the starting and ending vertices of τ, used for analyzing non-full-length transcripts.

Intron-Specific Metrics

- (introns): Number of introns inferred from another transcript $\tau' \in G$. An intron is considered if there is a splice junction of τ' located within an exon area of τ. Refer to “Examples” for details.
- (intron_ratio): Ratio of the weight of a splicing junction to the weight of adjacent junctions which is incident to a potential intron. This ratio quantifies the relative significance of splicing at that location. Refer to “Examples” for details.
- Endpoint Introns and Ratios (start_introns, end_introns, etc.): Counts and weight ratio of possible introns specifically at starting or ending exon areas.

Auxiliary Features

- (sample_size): Total number of samples in the dataset, used for normalization.
- (combined): Indicates if τ is outputted from the combined graph.

Examples

Here, we illustrate a couple of specific features. The first one is a boundary feature, quantifying the weights of junctions “overlooked” due to a starting or ending vertex. Consider the starting vertex v_1 in transcript τ assembled from G_0 in Supplementary Figure 1(left). We examine vertices in other graphs that span $R(v_1)$, such as v_3' in G_1 and v_3'' in G_2. For each such vertex we look for junctions as its in-edges; if none, we will check its preceding vertex (connected by adjacent edge) for junctions as in-edges, and so on. We calculate the sum of weights of found junctions as the feature. In this example, the feature value for v_1 is $w(e_1') + w(e_2'') + w(e_3'')$. This feature is expected to be informative in identifying false transcript boundaries and hence filtering incomplete transcripts.

The second example is an intron feature, calculated by comparing a candidate transcript τ is with another τ' assembled from the same graph. See Supplementary Figure 1(right). A potential intron of τ is considered if a splice junction e' of τ' falls within one or several consecutive vertices in τ. The feature, termed as “intron ratio”, is defined as the ratio of $w(e')$ over the minimum weight of adjacent edges (e.g., e_1 and e_2 in the example) of the potential intron in the graph. This ratio evaluates the relative significance of splicing events and is hence informative in identifying false intron-retentions.

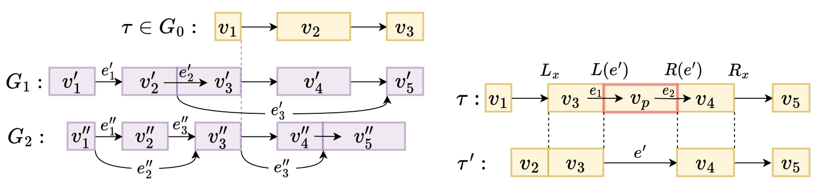

Supplementary Figure 1: Illustrative examples for two features. Left: A boundary feature. In G_1, e_1' is the first splice junction to the left of $R(v_1)$. Right: An intron feature. e' identifies v_p is a possible intron.

Supplementary Note 2: Thresholds for Precision-Recall Curves

TransMeta, Stringtie2-Merge and TACO do not include explicit coverage information in their output GTF files. Instead, these tools accept input parameters for minimum coverage, producing results corresponding to these discrete coverage thresholds. Conversely, PsiCLASS does report coverage in its output GTF; however, the reported values are not sufficiently accurate to validate transcript correctness, as indicated in Supplementary Figure 2. For PsiCLASS, in the main text, we used the internal voting coverage threshold (-vd option) to set different coverage levels. For all compared tools in Fig. 5-8, we draw the Precision-Recall Curves based on the discrete threshold values, connected by dashed lines. The coverage thresholds are specified as [0, 0.5, 1, 2, 5, 10, 20, 30, 50, 80, 100, 150, 200, 250, 300, 400, 500].

In the main text, we focus on Aletsch's scoring curve and PsiCLASS's voting curve instead of coverage. In Supplementary Figure 2, the coverage curves for Aletsch and PsiCLASS are presented in dotted lines across various coverage levels. For dataset BK-H1, Aletsch's coverage curve surpasses TransMeta's, while PsiCLASS's coverage correlation to precision is weak, yielding only around 60% precision as recall decreases. For SC-H1, Aletsch's coverage curve slightly underperforms compared to TransMeta but achieves high recall, allowing the scoring model to further identify reliable transcripts. PsiCLASS, however, with its narrow range of performance, achieves high precision but suffers from low recall.

Aletsch demonstrates competitive performance through its coverage curve. It initially generates a broad pool of candidate transcripts without heavily relying on heuristic parameters. A brute-force strategy to enumerate all possible paths is impractical due to its exponential time complexity and the risk of overwhelming the scoring model with negatively biased inputs. Aletsch aims to create an optimal set of candidate transcripts for the scoring model. As shown in the figure, there is a gap between coverage and scoring curves. Coverage, while important, is inadequate alone for scoring.

Supplementary Note 3: Comparison of Running Time and Memory

When multi-threading is available, we used 10 threads for running the tools. For single-sample assemblers like StringTie2 and Scallop2, we calculated the cumulative time across all samples. While Aletsch does not lead in CPU time, it demonstrates competitive performance. For example, in the case of the BK-H1 dataset, Aletsch completed its assembly in 229 minutes, compared to 589 minutes for TransMeta, 387 minutes for PsiCLASS. Regarding wall clock time, Aletsch was the fastest for three of the four bulk datasets examined, owing to its highly efficient multi-threading approach that significantly enhances parallelism. A complete comparison is available in Supplementary Table 4. Aletsch uses more memory than other approaches (Supplementary Table 5), yet its memory consumption remains within practical limits.

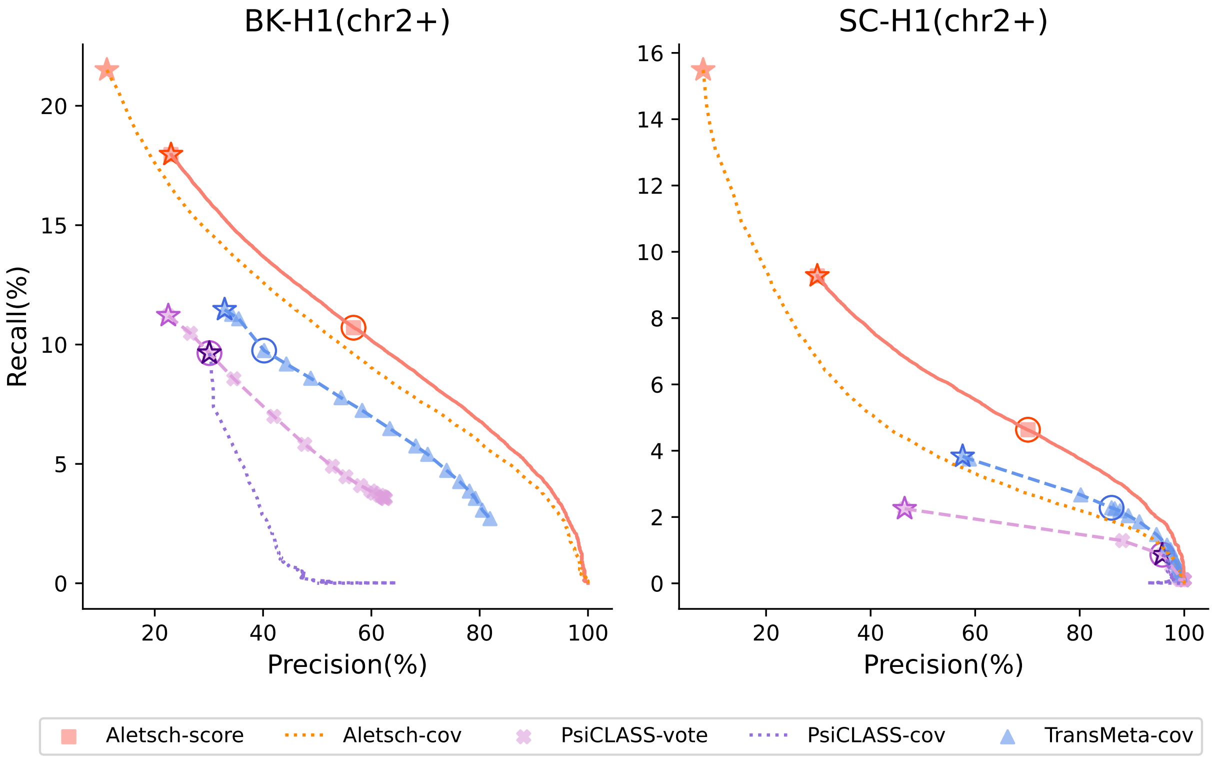

Supplementary Figure 2: Coverage curves of meta-assemblers on BK-H1 and SC-H1. Aletsch-cov and PsiCLASS-cov denote coverage curves for Aletsch and PsiCLASS, respectively. Aletsch-Chr1 model is used for Aletsch's scoring curve, Aletsch-score. PsiCLASS's voting curve, PsiCLASS-vote, is depicted by varying voting coverage thresholds. Circled points show default settings; starred points indicate filtering disabled.

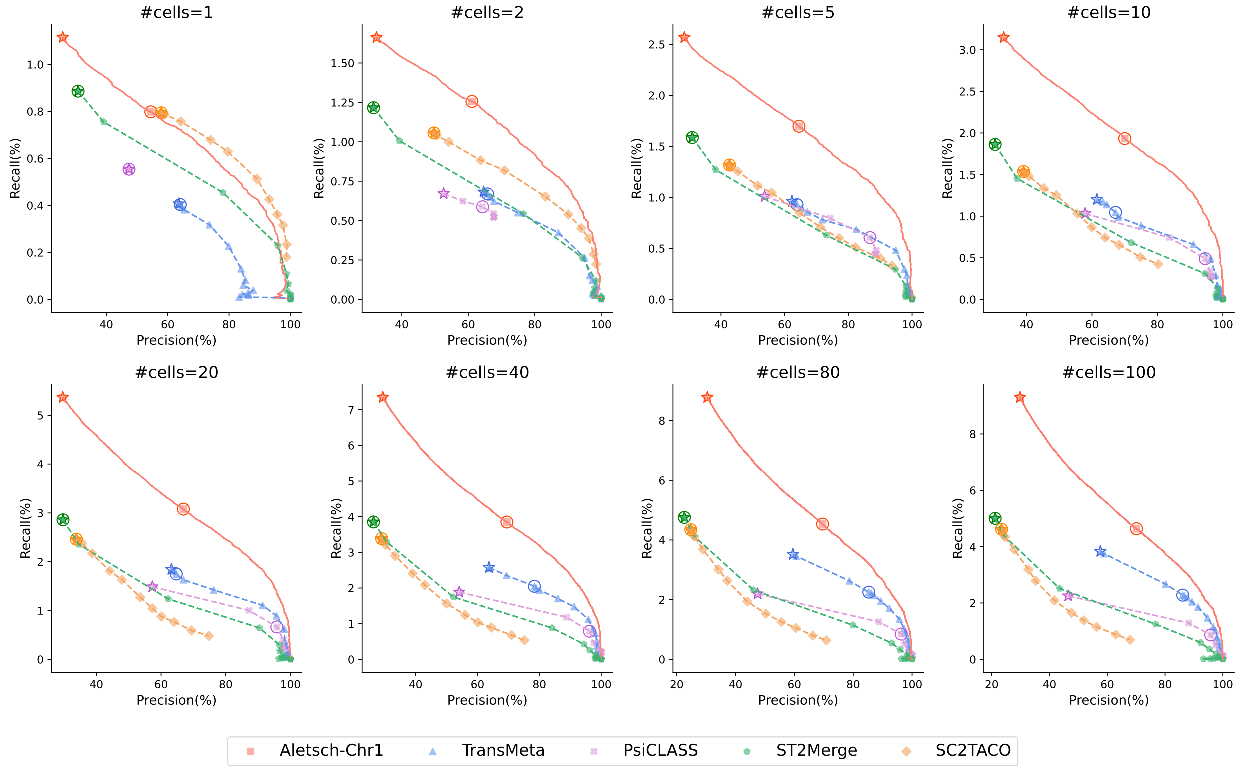

Supplementary Figure 3: How number of samples impact on meta-assembler performance. We evaluated the performance across varying cell counts (1, 2, 5, 10, 20, 40, 80, 100) using the SC-H1(Chr2+, 100 cells) dataset. Aletsch consistently outperforms competitors in multi-sample scenarios (more than 1 cell). In the single-sample scenario, SC2TACO leads, with Aletsch a close second—a predictable outcome given the scoring model's design for multi-sample analysis. With a single-sample as input, some features in the Aletsch's scoring model become invariant across candidate transcripts. TransMeta and PsiCLASS show inferior performance to single-sample assembler systems at a limited number of samples but improve as the number increases (≥ 5).

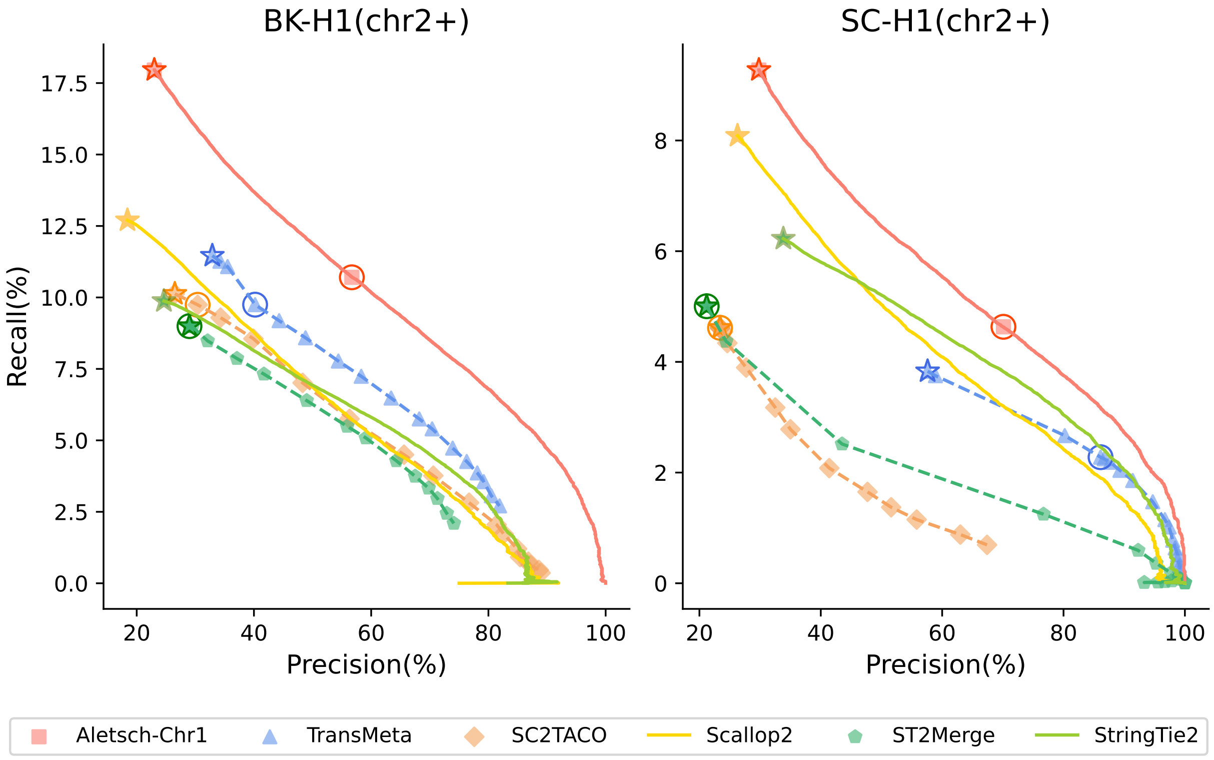

Supplementary Figure 4: Performance of merged input alignments on single-sample assemblers. Alignment files from the BK-H1/SC-H1 datasets are merged for input into single-sample assemblers Scallop2 and Stringtie2, depicted by solid bright green and yellow curves, respectively. Compared to single-sample assembler pipelines ST2Merge and SC2TACO, merging inputs—rather than outputs—significantly enhances the performance of single-sample assemblers, especially for single-cell datasets. Transcripts assembled from a single cell often remain fragmented, so it is important to use shared sample information to reconstruct complete transcripts. However, this strategy of merged inputs is not discussed in the main text, due to scalability concerns (e.g., Scallop2 took 10 hours to process merged BK-H1 alignments) and the ability of meta-assemblers to produce individual sample GTFs for downstream analysis. Additional steps are required for single-sample assemblers to assign transcripts from merged inputs back to specific samples.

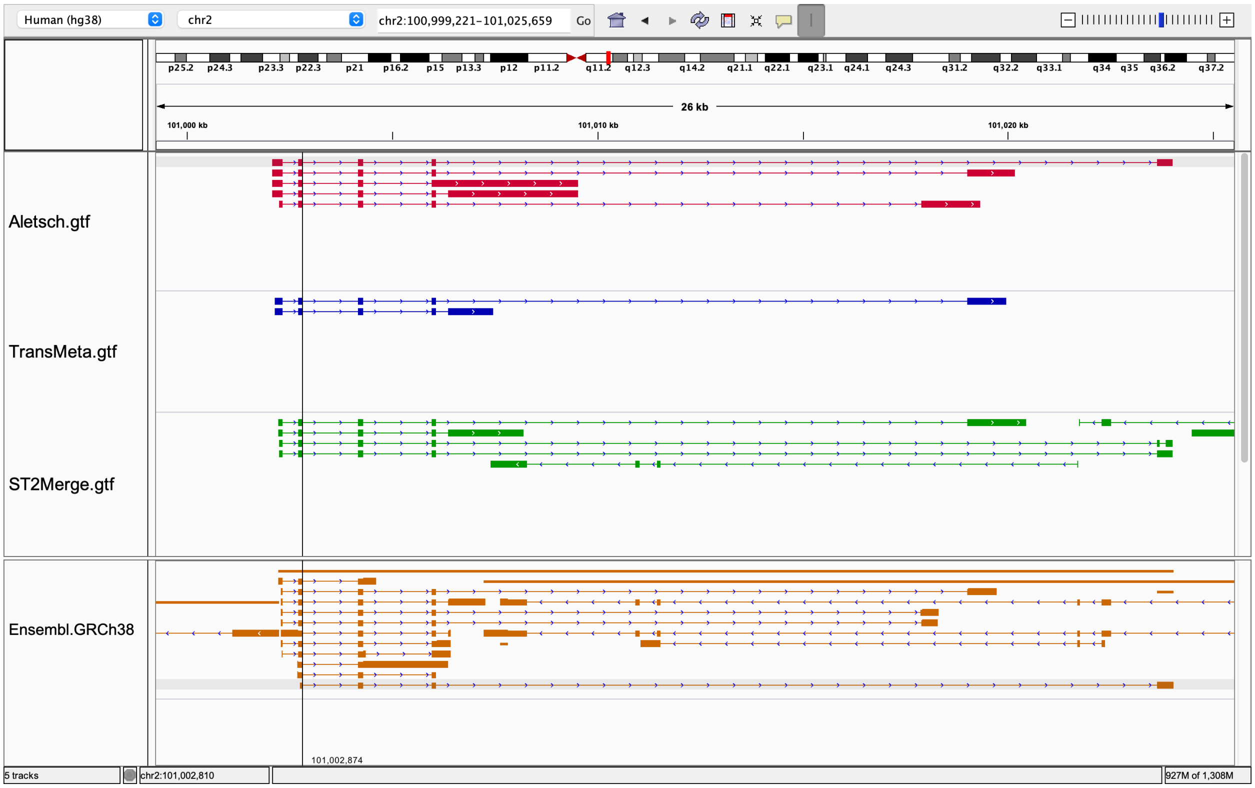

Supplementary Figure 5: Case study 1: performance comparison at gene RPL31. Within the SC-H1 dataset's RPL31 locus, Aletsch(red) identifies five transcripts, with four matching with the annotation(orange). The mismatched transcript is shaded in light grey, differing by an additional intron-chain at the left end compared to its closest annotated counterpart, also shaded in light grey. TransMeta(blue) generates 2 accurate transcripts, while ST2Merge(green) yields 5, with only 2 correct. This example demonstrates Aletsch's superior balance between precision and recall.

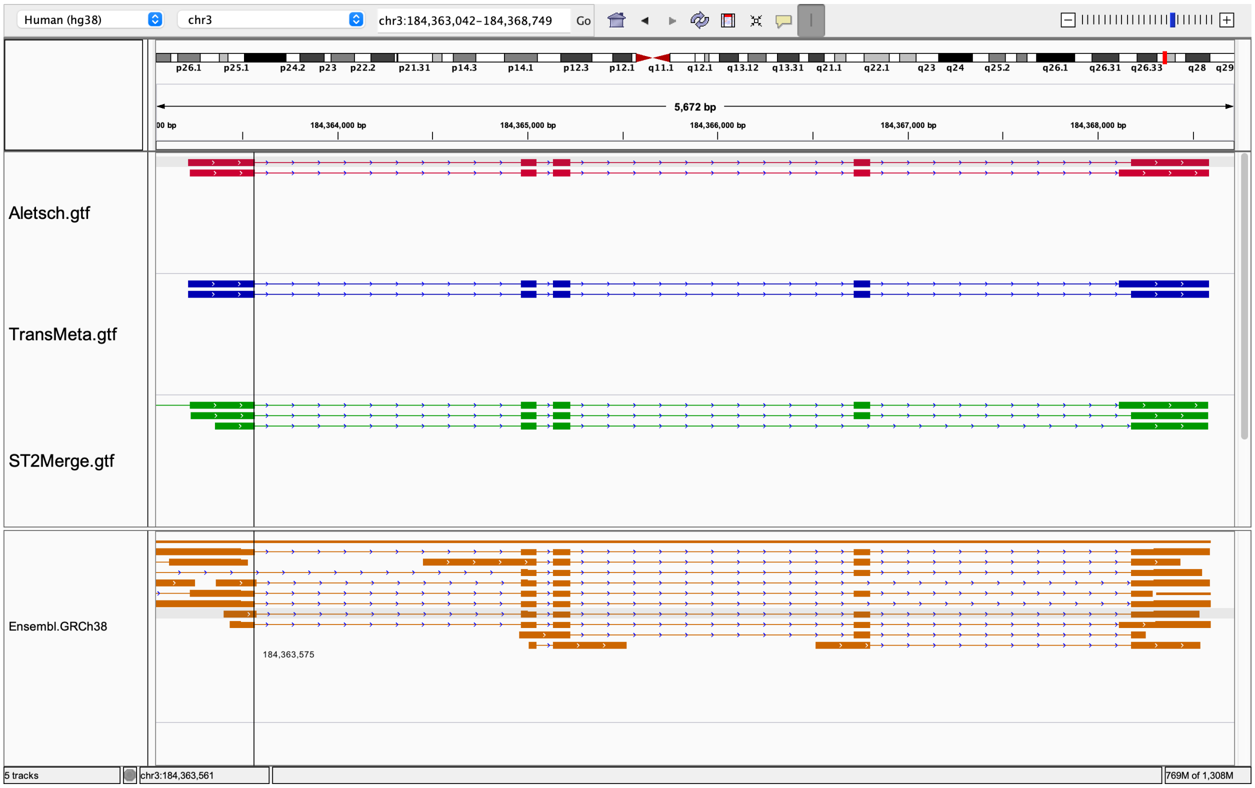

Supplementary Figure 6: Case Study 2: High-scored mismatch of Aletsch at gene POLR2H. Within the SC-H1 dataset at the POLR2H locus, Aletsch(red) predicts two transcripts; the top one, shaded in light grey, does not appear in the annotations(orange) and shows a minor deviation at the right boundary of the first exon. Despite receiving a high confidence score of 0.997, being detected in 96 out of 100 cells, and having support from TransMeta(blue) and ST2Merge(green), the biological authenticity of this potential novel transcript awaits validation through wet lab experiments.

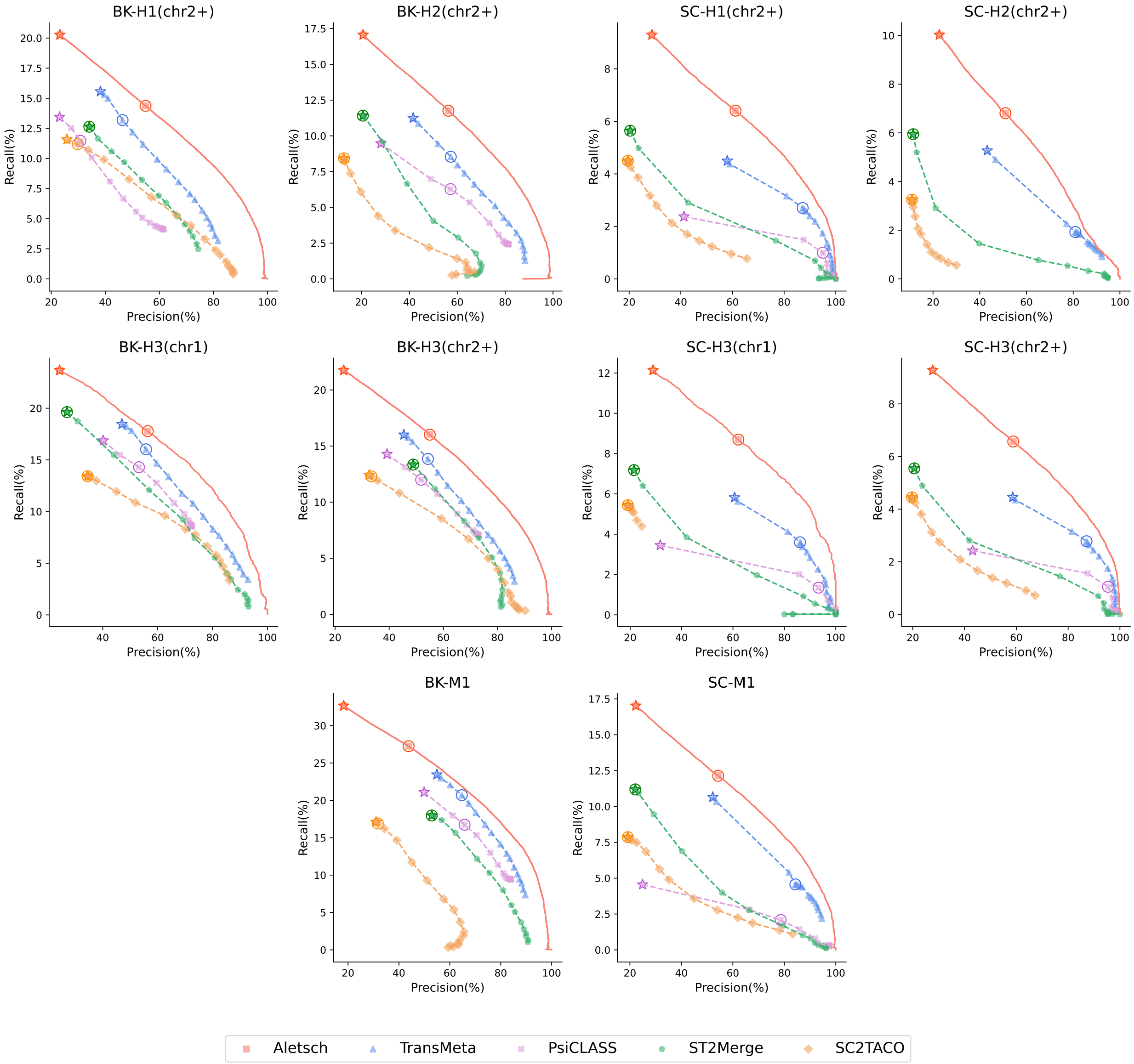

Supplementary Figure 7: Precision-recall curves evaluated on RefSeq annotation. Aletsch-Chr1 was used for G1 and G2 datasets; and Aletsch-ChrAll for G3. Aletsch-Chr1 and Aletsch-ChrAll were trained with labels according to the RefSeq annotation. PsiCLASS experienced segment-fault on SC-H2.

Supplementary Table 1: Comparison of pAUC (%, constrained by recall) between Aletsch and each other method. Aletsch-Chr1 was used for G1 and G2 datasets; Aletsch-ChrAll was used for G3 datasets. PsiCLASS experienced segment-fault on the SC-H2 dataset. Evaluation is against the RefSeq annotations.

Dataset	Aletsch vs. TransMeta			Aletsch vs. PsiCLASS			Aletsch vs. ST2Merge			Aletsch vs. SC2TACO		
	Alet.	Tran.	Δ%	Alet.	PsiC.	Δ%	Alet.	ST2M.	Δ%	Alet.	SC2T.	Δ%
BK-H1(chr2+)	9.61	7.60	26.4	7.56	3.74	102.0	8.64	5.69	52.0	9.97	6.81	46.4
BK-H2(chr2+)	8.36	6.82	22.7	6.08	4.04	50.5	9.51	5.04	88.8	7.45	2.72	173.6
SC-H1(chr2+)	3.95	3.60	9.7	2.22	1.86	19.0	5.10	3.00	70.2	3.47	1.31	165.3
SC-H2(chr2+)	3.46	2.95	17.0	N/A	N/A	N/A	4.73	1.88	151.2	2.34	0.43	442.2
BK-H3(chr1)	12.07	10.76	12.2	6.37	4.84	31.6	15.22	11.87	28.2	8.87	6.68	32.7
BK-H3(chr2+)	10.57	8.90	18.7	5.64	4.12	36.8	11.17	8.86	26.1	10.87	8.07	34.7
SC-H3(chr1)	5.17	4.65	11.2	3.24	2.59	25.2	6.66	3.74	78.1	0.94	0.23	307.0
SC-H3(chr2+)	3.93	3.60	9.2	2.26	1.92	17.7	5.03	2.91	72.7	3.48	1.35	157.3
BK-M1	13.20	12.19	8.3	9.74	8.01	21.5	15.61	12.84	21.6	15.65	8.70	79.9
SC-M1	7.19	6.37	12.9	4.16	2.91	43.2	9.58	5.65	69.7	6.23	2.88	116.4

Supplementary Table 2: Comparison of pAUC (%, constrained by precision) between Aletsch and each other method. Aletsch-Chr1 was used for G1 and G2 datasets; Aletsch-ChrAll was used for G3 datasets. PsiCLASS experienced segment-fault on the SC-H2 dataset. Evaluation is against the RefSeq annotations.

Dataset	Aletsch vs. TransMeta			Aletsch vs. PsiCLASS			Aletsch vs. ST2Merge			Aletsch vs. SC2TACO		
	Alet.	Tran.	Δ%	Alet.	PsiC.	Δ%	Alet.	ST2M.	Δ%	Alet.	SC2T.	Δ%
BK-H1(chr2+)	5.83	4.25	37.2	6.43	3.17	102.9	5.85	3.22	81.7	8.62	4.18	106.0
BK-H2(chr2+)	4.79	3.27	46.6	6.35	3.36	89.2	6.61	2.73	142.3	7.73	1.76	340.1
SC-H1(chr2+)	1.88	1.28	47.5	3.13	1.00	212.3	5.02	1.85	172.0	3.68	0.94	292.8
SC-H2(chr2+)	2.29	1.52	50.4	N/A	N/A	N/A	5.30	1.24	328.5	1.94	0.24	699.8
BK-H3(chr1)	6.54	5.26	24.5	5.73	4.27	34.1	10.92	7.33	48.9	8.54	4.92	73.6
BK-H3(chr2+)	5.56	4.18	32.9	5.33	3.69	44.3	4.53	2.88	57.3	8.30	4.31	92.5
SC-H3(chr1)	2.45	1.49	64.9	5.44	1.65	228.8	6.73	2.09	222.5	0.64	0.27	140.8
SC-H3(chr2+)	1.84	1.26	46.1	2.97	1.00	196.0	4.98	1.78	180.1	3.74	0.96	288.9
BK-M1	6.74	5.92	13.9	7.20	5.42	32.9	7.37	4.28	72.3	9.01	3.42	163.3
SC-M1	3.77	2.88	31.1	7.84	2.04	283.4	8.26	3.29	151.1	8.03	2.29	250.3

Supplementary Table 3: Isoform-level and gene-level analysis of assemblers. Evaluating multi-exon isoform precision (Precision) and true positives (#Intron-Chain, IC) via GffCompare, alongside true positives of canonical transcripts (#Canonical-IC) identified by the "Ensembl_canonical" tag in GENCODE annotation. Across both bulk dataset BK-H1 and single-cell dataset SC-H1, Aletsch consistently reports the highest #Canonical-IC, indicative of highest gene-level sensitivity. In SC-H1, TransMeta and PsiCLASS show a tendency to output confident gene locus representatives, resulting in a high #Canonical-IC to #IC ratio. In contrast, single-sample assembler systems, like ST2Merge and SC2TACO, generate more transcripts at the cost of precision, yet still capture a larger number of canonical transcripts due to their volume of predictions. Overall, Aletsch demonstrates a superior balance between isoform-level and gene-level sensitivity.

**Tool**	**BK-H1(chr2+)**			**SC-H1(chr2+)**		
	Precision(%)	#IC	#Canonical-IC	Precision(%)	#IC	#Canonical-IC
Aletsch	56.7	21797	11058	70.0	9426	5463
TransMeta	40.2	19821	9984	86.1	4632	3567
PsiCLASS	30.1	19605	10226	95.8	1756	1547
ST2Merge	29.0	18293	9922	21.2	10178	4833
SC2TACO	30.4	19811	10416	23.4	9386	4564

Supplementary Table 4: Comparison of running time (in minutes) across real datasets. The datasets were all aligned with STAR to genome GRCh38 (for human) and GRCm39 (for mouse). We report both CPU time and the Wall time. Where available, multithreading was configured with 10 threads. For single-sample assemblers (Scallop2 and StringTie2), cumulative time of all samples is reported.

**Dataset**	**Aletsch**		**TransMeta**		**PsiCLASS**		**ST2Merge**		**SC2TACO**	
	CPU	Wall	CPU	Wall	CPU	Wall	CPU	Wall	CPU	Wall
BK-H1	229	28	589	147	387	83	117	96	866	1367
BK-H2	853	89	1093	209	548	139	828	732	1132	1586
BK-H3	166	18	286	47	144	34	79	80	374	675
BK-M1	679	73	954	165	359	58	299	237	1579	2507
SC-H1&3	167	19	181	54	66	24	26	24	172	288
SC-H2	1210	142	505	184	N/A	N/A	90	40	476	623
SC-M1	424	48	241	41	106	23	60	54	389	590

Supplementary Table 5: Comparison of maximum memory usage (in GB) across real datasets. The datasets were all aligned with STAR to genome GRCh38 (for human) and GRCm39 (for mouse). Where available, multithreading was configured with 10 threads.

**Dataset**	**Aletsch**	**TransMeta**	**PsiCLASS**	**ST2MERGE**	**SC2TACO**
BK-H1	6.75	1.52	4.22	1.15	17.02
BK-H2	12.09	0.65	17.25	0.83	2.62
BK-H3	5.42	1.01	1.28	0.51	17.08
BK-M1	21.41	0.78	1.33	0.73	9.26
SC-H1&3	4.16	0.23	1.65	0.17	0.17
SC-H2	25.44	0.56	N/A	0.12	0.22
SC-M1	9.19	0.34	2.03	0.30	0.23

Supplementary Table 6: Accession IDs of BK-H1 dataset.

Accession ID
SRR534319
SRR534291
SRR545695
SRR387661
SRR307911
SRR545723
SRR315323
SRR307903
SRR315334
SRR534307

Supplementary Table 7: Accession IDs of BK-M1 dataset.

ERR1779355	ERR1779332	ERR1779372	ERR1779317
ERR1779333	ERR1779382	ERR1779363	ERR1779327
ERR1779353	ERR1779300	ERR1779349	ERR1779367
ERR1779344	ERR1779346	ERR1779350	ERR1779334
ERR1779379	ERR1779320	ERR1779309	ERR1779321
ERR1779370	ERR1779351	ERR1779301	ERR1779343
ERR1779449	ERR1779513	ERR1779451	ERR1779414
ERR1779444	ERR1779452	ERR1779491	ERR1779420
ERR1779435	ERR1779430	ERR1779502	ERR1779500
ERR1779457	ERR1779432	ERR1779487	ERR1779489
ERR1779417	ERR1779443	ERR1779503	ERR1779422
